# Age of 40 Years or Younger Is an Independent Risk Factor for Locoregional Failure in Early Breast Cancer: A Single-Institutional Analysis in Saudi Arabia

**DOI:** 10.1155/2012/370385

**Published:** 2012-04-01

**Authors:** Volker Rudat, Hamdan El-Sweilmeen, Elias Fadel, Iris Brune-Erber, Alaa Ahmad Nour, Zinaida Bushnag, Nidal Masri, Saleh Altuwaijri

**Affiliations:** ^1^Department of Radiation Oncology, Saad Specialist Hospital, P.O. Box 30353, Al Khobar 31952, Saudi Arabia; ^2^Department of Haematology and Oncology, Saad Specialist Hospital, P.O. Box 30353, Al Khobar 31952, Saudi Arabia; ^3^Department of Surgery, Saad Specialist Hospital, P.O. Box 30353, Al Khobar 31952, Saudi Arabia; ^4^Department of Pathology, Saad Specialist Hospital, P.O. Box 30353, Al Khobar 31952, Saudi Arabia; ^5^SAAD Research & Development Center, Saad Specialist Hospital, P.O. Box 30353, Al Khobar 31952, Saudi Arabia

## Abstract

*Background*. This study was undertaken to evaluate the impact of prognostic factors on the locoregional failure-free survival of early breast cancer patients. *Methods*. In this single-institutional study, 213 breast cancer patients were retrospectively analysed. Fifty-five of 213 patients were ≤40 years of age at diagnosis. The impact of patient- or treatment-related factors on the locoregional failure-free survival was assessed using the Kaplan-Meier method. The simultaneous impact of factors on the locoregional failure-free survival was assessed using the Cox proportional hazards regression analysis. *Results*. The median follow-up time of the censored patients was 22 months (mean 28 months, range 3–92 months). On univariate analysis, statistically significant factors for the locoregional failure-free survival were the age (≤40 versus >40 years), T stage (Tis, T0–2 versus T3-4), molecular tumor type (luminal A versus luminal B, Her2neu overexpression, or triple negative), and lymphovascular status (LV0 versus LV1). On multivariate analysis, age and T stage remained statistically significant. *Conclusions*. Being 40 years or younger has a statistically significant independent adverse impact on the locoregional failure-free survival of patients with early breast cancer.

## 1. Background

Approximately 3.7%–7.5% of the total number of breast cancer patients diagnosed each year in the US [1, 2] and Western Europe [3–5] are younger than 40 years. In Saudi Arabia, the proportion of breast cancer patients ≤40 years at diagnosis is dramatically larger with 25.1% [6].

Multiple retrospective series and subset analyses of larger randomized trials have shown that young patients with breast cancer have a poorer prognosis [7–16] compared to older age at diagnosis. Breast cancer patients ≤40 years tend to have more triple-negative and fewer luminal A and B breast cancers [17–19], tumors of higher grade, more extensive intraductal component, more lymphovascular invasion, more likely estrogen receptor- (ER-) negative tumors [20–23], and more often BRCA-1 or -2 germline mutations [13, 24–27]. Although young women do appear to have tumors with more aggressive biological characteristics, younger age has been shown in several studies to be an independent predictor of adverse outcome [18, 20, 22, 28–31]. Several current consensus guidelines have included age ≤35 years as an absolute indication for adjuvant systemic chemotherapy irrespective of other tumor characteristics [32–35]. More research is needed to optimize the treatment for this patient group [14, 36, 37]. Detailed data about prognostic factors and treatment outcome in breast cancer are scarce in Saudi Arabia and the Middle East. The purpose of this study was to characterize the breast cancer patients treated with curative intend of this tertiary care hospital in Saudi Arabia ≤40 years of age at diagnosis compared to >40 years, and to assess their prognosis.

## 2. Methods

Medical records were retrospectively reviewed of female breast cancer patients who consulted Saad Specialist Hospital between 2004 and 2011. Eligibility criteria for the analysis were histologically confirmed diagnosis of invasive breast cancer or cancer in situ, surgical treatment with breast conserving surgery or mastectomy with curative intent. Patients with distant metastases, synchronous, or metachronous cancer at diagnosis were excluded from the analysis. 

Staging procedures included complete history and physical examination, laboratory assessments, and diagnostic bilateral mammogram. Where indicated, ultrasonography of the breast and abdomen, chest radiograph, and radionuclide bone scan were performed. Selected patients received magnetic resonance imaging (MRI) of the breast, computerized tomography (CT), or positron emission tomography-computed tomography (PET-CT). Patients were presented and discussed in an interdisciplinary Tumor Board Meeting, and a treatment recommendation was generated usually based on the guidelines of the National Comprehensive Cancer Network (NCCN). 

Breast conserving surgery (BCS) consisted of wide local excision or lumpectomy and axillary dissection or sentinel lymph node biopsy in selected patients. After modified radical mastectomy, in selected patients breast reconstruction with TRAM-flap or latissimus dorsi-flap was performed. Surgery was followed by chemotherapy and hormonal therapy where indicated. Dependent on the T status, N status, hormone receptor status, age (≤35 years versus >35 years), and menopausal status, four cycles of Adriamycin/Cyclophosphamide (AC) or six cycles of Cyclophosphamide/Methotrexate/5-FU (CMF) were prescribed for node-negative patients, and four cycles of AC followed by four cycles of paclitaxel or, alternatively, three cycles of 5-FU/Epirubicin/Cyclophosphamide (FEC) followed by three cycles of docetaxel for node positive patients. Endocrine therapy using tamoxifen or aromatase inhibitors was prescribed where indicated. Herceptin was added according to the Her2neu status and prescribed for at least one year. Triple negative patients were usually treated with four cycles of AC followed by four cycles of paclitaxel. In selected patients neoadjuvant chemotherapy was applied. 

Postoperative radiotherapy was performed in all patients after BCS. A total dose of 50.4 Gy in 28 fractions was prescribed, followed by a boost of 10 Gy in 5 fractions in all patients younger than 50 years. Postmastectomy radiotherapy of the chest wall was given in patients with at least one positive locoregional lymph node. The prescribed dose was 50 Gy in 25 fractions. Usually three-dimensional conformal radiotherapy (3D-CRT) using opposed tangential beam was applied for the treatment of the whole breast or the chest wall. In selected patients, intensity modulated radiotherapy (IMRT) was used to reduce the dose volume of the heart and lung [38]. 

Follow-up examinations were scheduled every three months in the first year, then every six months for 4 years. 

Breast cancer was classified according to the International Union Against Cancer (UICC), with group clinical and pathological staging according to the American Joint Committee on Cancer (AJCC, 6th edition). 

Data were entered into a computerized database (MS Access 2010) and analysed using a statistical software package (SPSS 19). 

### 2.1. Immunohistochemistry

Sections with a thickness of four *μ*m were cut from paraffin blocks and used for immunohistochemical staining using the iVIEW DAB detection kit on BenchMark autostainer (Ventana, Tucson, AZ, USA). The clones of antibodies SP1, 1E2, and 4B5 were used to evaluate the ER-a, PR, and Her2neu status. The Allred scoring system was used to assess the ER and PR status [39]. In summary, a total Allred score was obtained by the summation of proportion score (PS) and intensity score (IS). PS is assigned depending on the proportion of positive cells (0 = none; 1 <1%; 2 = 1% -<1/10; 3 = 1/10 -<1/3; 4 = 1/3 -<2/3; 5 ≥2/3), IS (0 = none; 1 = weak; 2 = intermediate; 3 = strong). A total score of 2 or more was considered as positive; scores 0 and 1 were considered negative. 

The American Society of Clinical Oncology/College of American Pathologists (ASCO/CAP) guideline recommendations were used to evaluate the Her2neu status [40]. Briefly, score 0 indicates no staining in invasive tumor cells. Score +1 indicates weak incomplete membrane staining in any proportion of invasive tumor cells or weak complete membrane staining in <10% of cells. Score +2 indicates complete membrane staining in nonuniform or weak but with obvious circumferential distribution in ≥10% of cells, or intense complete membrane staining in ≤30% of tumor cells. Score +3 indicates uniform intense membrane staining of >30% of invasive tumor cells. Scores 0 and +1 were considered negative; +2 equivocal; and +3 positive. 

Gene expression profiling studies have shown that immunohistochemistry of paraffin sections is a reliable surrogate for molecular classification of invasive breast cancers [41–46]. Based on this finding, patients of this study were categorized as follows: luminal A (ER+, PR+, Her2neu−), luminal B (ER+ and/or PR+, Her2neu+), Her2neu overexpressing (ER−, PR−, Her2neu+), and triple negative (ER−, PR−, Her2neu−). 

### 2.2. Statistical Analysis

The *Z*-test was used to test for statistically significant different proportions concerning disease- and treatment-related factors of patients ≤40 years versus >40 years. 

The locoregional failure-free survival was estimated using the Kaplan-Meier method, and patient groups were compared using the log rank test. The locoregional failure-free survival was defined as the time between diagnosis and locoregional failure or death of any cause. Patients who have not experienced a locoregional failure were censored at the time of their last follow-up. The simultaneous relationship of multiple prognostic factors to locoregional failure was assessed using Cox's proportional hazard regression analysis. The regression coefficients were estimated by the maximum likelihood method, and model selection was performed by a stepwise strategy using the likelihood ratio test. A 5% significance level was used and all tests are two-sided. No correction for multiple testing was used. 

## 3. Results

The proportion of patients ≤40 years of all breast cancer patients who consulted Saad Specialist Hospital was 22.6%, which is very much in accordance to the proportion of breast cancer patients ≤40 years published by the Cancer Incidence and Survival Report Saudi Arabia 2007 of 25.1% [6]. Two hundred and thirteen of all breast cancer patients met the eligibility criteria of this study and were analysed. Of those, 158 patients were >40 years of age at diagnosis and 55 patients ≤40 years. 

The patient and treatment characteristics are demonstrated in Table 1. The median follow-up time of the censored patients was 22 months (mean 28 months, range 3–92 months). 

Patients of ≤40 years at diagnosis exhibited statistically significantly less frequently the tumor type luminal A, and statistically significantly more frequently the tumor type triple negative compared to patients >40 years (Table 1). In addition, the body mass index (BMI) and the menopausal status were significantly different in the two age groups (Table 1). The mean BMI (standard deviation) of all patients was 31.1 (6.0), of patients ≤40 years 28.3 (5.2), and for patients >40 years 32.1 (6.0). 

**Figure 1:**
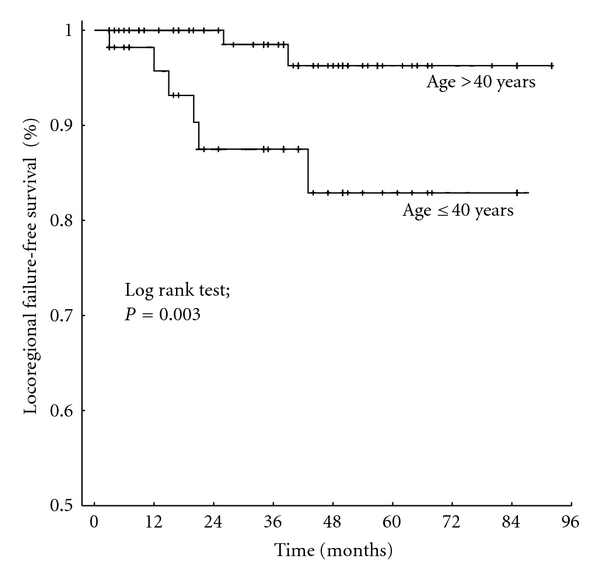
Locoregional failure-free survival of breast cancer patients ≤40 years versus >40 years of age at diagnosis.

**Figure 2:**
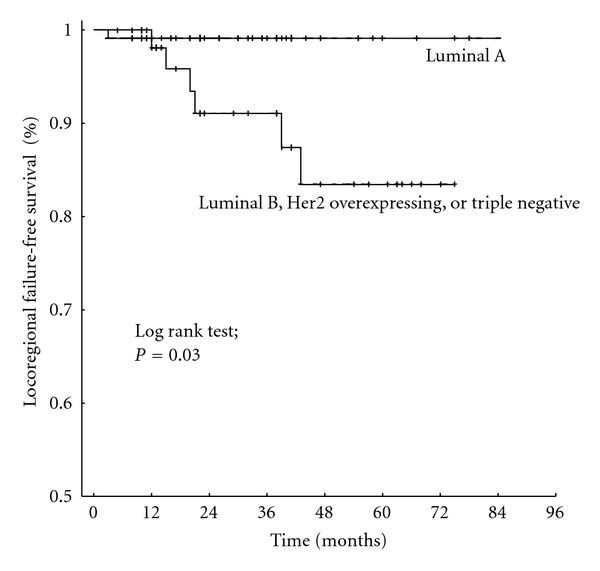
Locoregional failure-free survival of breast cancer patients with luminal A type of tumor versus luminal B, Her2neu overexpression, or triple negative.

On univariate analysis, the age (≤40 versus >40 years), T stage (Tis, T0–2 versus T3-4), molecular tumor type (luminal A versus luminal B, Her2neu overexpression, triple negative), and lymphovascular status (LV0 versus LV1) had a significant impact on the locoregional failure-free survival (Table 2, Figures 1 and 2). The N status (N0 versus N+) showed a statistical trend of an association with the locoregional failure-free survival (*P* = 0.13). On multivariate analysis, the age and T stage remained statistically significant (Table 3).

## 4. Discussion

In Saudi Arabia, the proportion of breast cancer patients ≤40 years of age at diagnosis is about three times larger than generally reported in the West (25.1% versus 3.7%–7.5%). According to the US Census Bureau [47], the proportion of females ≤40 years of the Saudi Arabian population is only 1.5 times larger than that of the US population (80.4% versus 52.2%; year 2010), suggesting that the larger proportion of breast cancer patients ≤40 years in Saudi Arabia may not be fully explained by the different age structures of the two populations. This observation and also the fact that some studies performed in Asia and Africa did not find a different prognosis of younger breast cancer patients compared to the older counterparts [48, 49] suggest that regional differences may exist concerning the biology and prognosis of young breast cancer patients. Detailed clinicopathological and prognostic data are scarce in Saudi Arabia and the Middle East. 

Our data show that young age is an independent negative prognostic factor for the locoregional control of breast cancer patients in Saudi Arabia. The same finding has been reported by another retrospective single-institutional study in Saudi Arabia [50]. Our findings are compatible with the notion that breast cancer arising in a younger host is a unique entity characterized not only by adverse prognostic features, but also by a diverse underlying biology against which novel therapeutics should be targeted [1, 31]. Several current consensus guidelines have included age ≤35 years as an absolute indication for adjuvant systemic chemotherapy irrespective of other tumor characteristics [32–35]. The observation of a higher locoregional recurrence rate after BCS compared to mastectomy in patients ≤35 years of age in some studies [20, 21, 28, 51–56] raised the question about the optimal surgical approach of this patient group. However, a recently published large population-based analysis consisting of 1,453 early breast cancer patients ≤40 years showed that the 10-year overall survival was not impaired after BCS compared to mastectomy [57]. In line with this observation, no difference of the two-year locoregional failure-free survival was detected in our study after BCS or mastectomy (*P* = 0.59). A pooled analysis of four EORTC randomized controlled trials revealed that tumor size, nodal status, and molecular tumor subtype were independent prognostic factors for overall survival of the subgroup of young breast cancer patients [58]. The authors concluded that future treatment guidelines concerning young breast cancer patients should be refined based upon tumor characteristics, probably derived from microarray driven translational research projects, and not based upon age alone [59–61]. In our study, the molecular tumor subtype had a prognostic relevance for all patients on univariate analysis, but lost its significance on multivariate analysis. 

Breast cancer in young women is probably the result of a complex interaction between genetic, environmental, and nongenetic patient related factors [24, 25, 27, 31, 62–68]. However, no significant difference of the family history was found between breast cancer patients ≤40 years and >40 years in our study. A striking difference to representative data from North America or Europe was the significantly higher BMI of our study patients. The mean BMI (standard deviation) of our study population was 31.1 (6.0) compared to 24.8 (4.4) in North America [69]) and 25.5 (4.5) in Europe [70]. A closer look revealed that the main difference was confined to the subgroup of young patients. In our study, the proportion of patients ≤40 years with a BMI of ≤25, 25–30, and >30 was about 33% for each category, whereas in the western studies the corresponding proportions ranged from 57.0%–73.2%; 19.4%–27.5%, and 12.1%–17.1%, respectively [71–75]. The differences of the BMI of older patients compared to the West were much smaller (our study: 58.5%, 30.5%, and 11.0%; western studies: 41.6%–52.9%, 28.7–37.5%, and 11.5%–20.9% [74, 75]). 

Studies have shown that the BMI is associated with poor prognosis in both premenopausal [72–74, 76, 77] and postmenopausal patients [74–80]. In addition, the BMI has been shown to be associated with an increased breast cancer risk in postmenopausal women [69, 70, 81]. In premenopausal women, a protective or no effect of the BMI on the breast cancer risk has been observed [70, 71, 82–86]. In contrast, several studies considering multiple surrogate markers for obesity have also reported an association of obesity with an increased risk to develop breast cancer in premenopausal women [69, 87, 88]. Although the association between body weight and breast cancer appears to be complex, it represents an interesting factor to be evaluated in future studies concerning breast cancer in Saudi Arabia.

## 5. Conclusions

Patients ≤40 years exhibited more often triple negative and less frequently luminal A tumors compared to patients >40 years. However, multivariate analysis revealed age ≤40 years as an independent adverse prognostic factor for the locoregional failure-free survival of breast cancer patients in Saudi Arabia. 

## Figures and Tables

**Table 1:** Comparison of clinical and pathological characteristics of patients ≤40 years and >40 years of age at diagnosis.

Characteristic	Age (years)				*P* value
	≤40		>40		
	*n*	%	*n*	%	
Body mass index					
<25	14	25.5	13	8.2	≤0.05
25–29	14	25.5	36	22.8	n.s.
≥30	14	25.5	69	43.7	≤0.05
Unknown	13	23.6	40	25.3	n.s.

Menopausal status					
Premenopausal	54	98.2	75	47.5	≤0.05
Postmenopausal	1	1.8	86	52.5	≤0.05

Family history					
No cancers in blood relatives	23	41.8	57	36.1	n.s.
Other than breast cancer in at least one blood relative	1	1.8	4	2.5	n.s.
Other than breast cancer in at least one first degree relative	6	10.9	8	5.1	n.s.
Breast cancer or ovarian cancer in at least one blood relative	4	7.3	13	8.2	n.s.
Breast cancer or ovarian cancer in at least one first degree relative	2	3.6	19	12.0	n.s.
Unknown	19	34.5	57	36.1	n.s.

Histology					
Invasive ductal	49	89.1	143	90.5	n.s.
Invasive lobular	2	3.6	12	7.6	n.s.
DCIS	3	5.5	3	1.9	n.s.
LCIS	1	1.8	0	0	n.a.

T stage					
Tis	4	7.3	3	1.9	n.s.
T0	1	1.8	0	0.0	n.a.
T1	16	29.1	55	34.8	n.s.
T2	22	40.0	59	37.3	n.s.
T3	4	7.3	27	17.1	n.s.
T4	6	10.9	10	6.3	n.s.
Tx	2	3.6	4	2.5	n.s.

N stage					
N0	18	32.7	59	37.3	n.s.
N1	17	30.9	46	29.1	n.s.
N2	11	20.0	23	14.6	n.s.
N3	6	10.9	26	16.5	n.s.
Nx	3	5.5	4	2.5	n.s.

Stage					
0	4	7.3	3	1.9	n.s.
I	6	10.9	35	22.2	n.s.
II	19	34.5	56	35.4	n.s.
III	23	41.8	59	37.3	n.s.
Unknown	3	5.5	5	3.2	n.s.
Grading					
G1	2	3.6	14	8.9	n.s.
G2	17	30.9	47	29.7	n.s.
G3	22	40.0	71	44.9	n.s.
Gx	14	25.5	26	16.5	n.s.

Lymphovascular invasion (LV)					
LV0	17	30.9	50	31.6	n.s.
LV1	15	27.3	53	33.5	n.s.
Unknown	23	41.8	55	34.8	n.s.

Type of surgery					
Breast conserving surgery	22	40.0	70	44.3	n.s.
Mastectomy	33	60.0	88	55.7	n.s.

Neoadjuvant chemotherapy					
No	46	83.6	137	86.7	n.s.
Yes	9	16.4	21	13.3	n.s.

Residual tumor (R) status					
R0	41	74.5	122	77.2	n.s.
R1	3	5.5	12	7.6	n.s.
Unknown	11	20.0	24	15.2	n.s.

Estrogen receptor (ER) status					
ER negative	17	30.9	43	27.2	n.s.
ER positive	33	60.0	106	67.1	n.s.
Unknown	5	9.1	9	5.7	n.s.

Progesterone receptor (PR) status					
PR negative	23	41.8	52	32.9	n.s.
PR positive	27	49.1	97	61.4	n.s.
Unknown	5	9.1	9	5.7	n.s.

Her2neu status					
Her2neu negative	33	60.0	115	72.8	n.s.
Her2neu positive	12	21.8	29	18.4	n.s.
Unknown	10	18.2	14	8.9	n.s.

Tumor subtype					
Luminal A	20	36.4	94	59.5	≤0.05
Luminal B	9	16.4	17	10.8	n.s.
Her2 overexpressing	3	5.5	12	7.6	n.s.
Triple negative	14	25.5	21	13.3	≤0.05
Unknown	9	16.4	14	8.9	n.s.

**Table 2:** Results of the Kaplan-Meier analysis.

Factor	*n*	2-year locoregional failure-free survival	95% CI	*P* value
Age				0.005
Age ≤40	54	0.86	0.75–0.98	
Age >40	158	0.98	0.95–1.00	

Menopausal status				0.52
Premenopausal	129	0.99	0.97–1.00	
Postmenopausal	84	0.97	0.91–1.00	

BMI				0.31
BMI <30	72	0.95	0.88–1.00	
BMI ≥30	83	0.97	0.92–1.00	

T stage				0.03
Tis, T0–2	159	0.98	0.95–1.00	
T3-4	47	0.89	0.78–1.00	

N stage				0.13
N0	77	1.00	1.00-1.00	
N+	128	0.94	0.88–1.00	

Grading				0.27
G1-2	80	1.00	1.00-1.00	
G3	93	0.93	0.86–1.00	

Tumor subtype				0.03
Luminal A	75	0.91	0.83–0.99	
Others*	114	0.99	0.97–1.00	

Lymphovascular status				0.02
LV0	67	1.00	1.00-1.00	
LV1	68	0.90	0.79–1.00	

Type of surgery				0.59
Mastectomy	121	0.96	0.91–1.00	
BCS	91	0.97	0.92–1.00	

Residual tumor status				0.56
R0	163	0.97	0.93–1.00	
R1	15	1.00	1.00-1.00	

Abbreviations. BMI: body mass index; *: luminal B, Her2 overexpressing, or triple negative; BCS: breast conserving surgery; CI: confidence interval.

**Table 3:** Results of Cox proportional hazards regression analysis.

Variable	*P* value	Estimated relative hazard	95% CI for relative hazard
Age	0.01	0.13	0.03–0.66
T stage	0.05	4.06	1.01–16.3

## Abbreviations

AC: Adriamycin/cyclophosphamide
BCS: Breast conserving surgery
BMI: Body mass index
CMF: Cyclophosphamide/methotrexate/5-FU
ER: Estrogen receptor
FEC: 5-FU/Epirubicin/cyclophosphamide
IS: Intensity score
PR: Progesterone receptor
PS: Proportion score.

## References

[1] Anders CK, Johnson R, Litton J, Phillips M, Bleyer A (2009). Breast cancer before age 40 years. *Seminars in Oncology*.

[2] Winchester DP (1996). Breast cancer in young women. *Surgical Clinics of North America*.

[3] http://info.cancerresearchuk.org.

[4] http://www.ikcnet.nl/.

[5] http://www.rki.de/cln_162/nn_204124/DE/Content/GBE/DachdokKrebs/KID/kid__node.html?__nnn=true.

[6] http://www.scr.org.sa/reports/SCR2007.pdf.

[7] Zabicki K, Colbert JA, Dominguez FJ (2006). Breast cancer diagnosis in women ≤ 40 versus 50 to 60 years: increasing size and stage disparity compared with older women over time. *Annals of Surgical Oncology*.

[8] Bollet MA, Sigal-Zafrani B, Mazeau V (2007). Age remains the first prognostic factor for loco-regional breast cancer recurrence in young (<40 years) women treated with breast conserving surgery first. *Radiotherapy and Oncology*.

[9] Aryandono T, Harijadi, Soeripto (2006). Breast cancer in young women: prognostic factors and clinicopathological features. *Asian Pacific Journal of Cancer Prevention*.

[10] Jayasinghe UW, Taylor R, Boyages J (2005). Is age at diagnosis an independent prognostic factor for survival following breast cancer?. *ANZ Journal of Surgery*.

[11] Foo CS, Su D, Chong CK (2005). Breast cancer in young Asian women: study on survival. *ANZ Journal of Surgery*.

[12] Han W, Kim SW, Park IA (2004). Young age: an independent risk factor for disease-free survival in women with operable breast cancer. *BMC Cancer*.

[13] Colleoni M, Rotmensz N, Robertson C (2002). Very young women (<35 years) with operable breast cancer: features of disease at presentation. *Annals of Oncology*.

[14] Aebi S, Gelber S, Castiglione-Gertsch M (2000). Is chemotherapy alone adequate for young women with oestrogen-receptor-positive breast cancer?. *The Lancet*.

[15] Fredholm H, Eaker S, Frisell J, Holmberg L, Fredriksson I, Lindman H (2009). Breast cancer in young women: poor survival despite intensive treatment. *PLoS One*.

[16] Voogd AC, Nielsen M, Peterse JL (2001). Differences in risk factors for local and distant recurrence after breast-conserving therapy or mastectomy for stage I and II breast cancer: pooled results of two large European randomized trials. *Journal of Clinical Oncology*.

[17] Carey LA, Perou CM, Livasy CA (2006). Race, breast cancer subtypes, and survival in the Carolina Breast Cancer Study. *JAMA*.

[18] Cancello G, Maisonneuve P, Rotmensz N (2010). Prognosis and adjuvant treatment effects in selected breast cancer subtypes of very young women (<35 years) with operable breast cancer. *Annals of Oncology*.

[19] Bauer KR, Brown M, Cress RD, Parise CA, Caggiano V (2007). Descriptive analysis of estrogen receptor (ER)-negative, progesterone receptor (PR)-negative, and HER2-negative invasive breast cancer, the so-called triple-negative phenotype: a population-based study from the California Cancer Registry. *Cancer*.

[20] Nixon AJ, Neuberg D, Hayes DF (1994). Relationship of patient age to pathologic features of the tumor and prognosis for patients with stage I or II breast cancer. *Journal of Clinical Oncology*.

[21] Kurtz JM, Jacquemier J, Amalric R (1990). Why are local recurrences after breast-conserving therapy more frequent in younger patients?. *Journal of Clinical Oncology*.

[22] Albain KS, Allred DC, Clark GM (1994). Breast cancer outcome and predictors of outcome: are there age differentials?. *Journal of the National Cancer Institute. Monographs*.

[23] Leborgne F (1995). Breast conservation treatment of early stage breast cancer: patterns of failure. *International Journal of Radiation Oncology Biology Physics*.

[24] Choi DH, Lee MH, Dale AE, Carter D, Haffty BG (2004). Incidence of BRCA1 and BRCA2 mutations in young Korean breast cancer patients. *Journal of Clinical Oncology*.

[25] Loman N, Johannsson O, Kristoffersson U, Olsson H, Borg A (2001). Family history of breast and ovarian cancers and *BRCA1* and *BRCA2* mutations in a population-based series of early-onset breast cancer. *Journal of the National Cancer Institute*.

[26] Maggard MA, O’Connell JB, Lane KE, Liu JH, Etzioni DA, Ko CY (2003). Do young breast cancer patients have worse outcomes?. *Journal of Surgical Research*.

[27] De Sanjosé S, Léoné M, Bérez V (2003). Prevalence of *BRCA1* and *BRCA2* germline mutations in young breast cancer patients: a population-based study. *International Journal of Cancer*.

[28] Oh JL, Bonnen M, Outlaw ED (2006). The impact of young age on locoregional recurrence after doxorubicin-based breast conservation therapy in patients 40 years old or younger: how young is “young”?. *International Journal of Radiation Oncology Biology Physics*.

[29] De la Rochefordiere A, Asselain B, Campana F (1993). Age as prognostic factor in premenopausal breast carcinoma. *The Lancet*.

[30] Matthews RH, McNeese MD, Montague ED, Oswald MJ (1988). Prognostic implications of age in breast cancer patients treated with tumorectomy and irradiation or with mastectomy. *International Journal of Radiation Oncology Biology Physics*.

[31] Anders CK, Hsu DS, Broadwater G (2008). Young age at diagnosis correlates with worse prognosis and defines a subset of breast cancers with shared patterns of gene expression. *Journal of Clinical Oncology*.

[32] De Bock GH, Van Der Hage JA, Putter H, Bonnema J, Bartelink H, Van De Velde CJ (2006). Isolated loco-regional recurrence of breast cancer is more common in young patients and following breast conserving therapy: long-term results of European Organisation for Research and Treatment of Cancer studies. *European Journal of Cancer*.

[33] Elkhuizen PHM, Voogd AC, Van Den Broek LCJM (1999). Risk factors for local recurrence after breast-conserving therapy for invasive carcinomas: a case-control study of histological factors and alterations in oncogene expression. *International Journal of Radiation Oncology Biology Physics*.

[34] Goldhirsch A, Gelber RD, Yothers G (2001). Adjuvant therapy for very young women with breast cancer: need for tailored treatments. *Journal of the National Cancer Institute. Monographs*.

[35] Goldhirsch A, Glick JH, Gelber RD, Coates AS, Hans-Jörg S (2001). Meeting highlights: international consensus panel on the treatment of primary breast cancer. *Journal of Clinical Oncology*.

[36] Balduzzi A, Cardillo A, D’Alessandro C, Colleoni M (2007). Adjuvant treatment for young women with early breast cancer. *Minerva Ginecologica*.

[37] Curigliano G, Rigo R, Colleoni M (2004). Adjuvant therapy for very young women with breast cancer: response according to biologic and endocrine features. *Clinical Breast Cancer*.

[38] Rudat V, Aziz Alaradi A, Mohamed A, AI-Yahya K, Altuwaijri S (2011). Tangential beam IMRT versus tangential beam 3D-CRT of the chest wall in postmastectomy breast cancer patients: a dosimetric comparison. *Radiation Oncology*.

[39] Allred DC, Harvey JM, Berardo M, Clark GM (1998). Prognostic and predictive factors in breast cancer by immunohistochemical analysis. *Modern Pathology*.

[40] Wolff AC, Hammond MEH, Schwartz JN (2007). American Society of Clinical Oncology/College of American Pathologists guideline recommendations for human epidermal growth factor receptor 2 testing in breast cancer. *Archives of Pathology and Laboratory Medicine*.

[41] Nielsen TO, Hsu FD, Jensen K (2004). Immunohistochemical and clinical characterization of the basal-like subtype of invasive breast carcinoma. *Clinical Cancer Research*.

[42] Abd El-Rehim DM, Ball G, Finder SE (2005). High-throughput protein expression analysis using tissue microarray technology of a large well-characterised series identifies biologically distinct classes of breast cancer confirming recent cDNA expression analyses. *International Journal of Cancer*.

[43] Abd El-Rehim DM, Pinder SE, Paish CE (2004). Expression of luminal and basal cytokeratins in human breast carcinoma. *Journal of Pathology*.

[44] Brenton JD, Carey LA, Ahmed A, Caldas C (2005). Molecular classification and molecular forecasting of breast cancer: ready for clinical application?. *Journal of Clinical Oncology*.

[45] Van de Rijn M, Perou CM, Tibshirani R (2002). Expression of cytokeratins 17 and 5 identifies a group of breast carcinomas with poor clinical outcome. *American Journal of Pathology*.

[46] Tamimi RM, Baer HJ, Marotti J (2008). Comparison of molecular phenotypes of ductal carcinoma in situ and invasive breast cancer. *Breast Cancer Research*.

[47] http://www.censusscope.org/us/chart_age.html.

[48] Khanfir A, Frikha M, Kallel F (2006). Breast cancer inyoung women inthesouth ofTunisia. *Cancer/Radiotherapie*.

[49] Chia KS, Du WB, Sankaranarayanan R (2004). Do younger female breast cancer patients have a poorer prognosis? Results from a population-based survival analysis. *International Journal of Cancer*.

[50] Elkum N, Dermime S, Ajarim D (2007). Being 40 or younger is an independent risk factor for relapse in operable breast cancer patients: the Saudi Arabia experience. *BMC Cancer*.

[51] Recht A, Connolly JL, Schnitt SJ (1988). The effect of young age on tumor recurrence in the treated breast after conservative surgery and radiotherapy. *International Journal of Radiation Oncology Biology Physics*.

[52] Coulombe G, Tyldesley S, Speers C (2007). Is mastectomy superior to breast-conserving treatment for young women?. *International Journal of Radiation Oncology Biology Physics*.

[53] Kurtz JM, Amalric R, Brandone H, Ayme Y, Spitalier JM (1989). Local recurrence after breast-concerving surgery and radiotherapy. *Helvetica Chirurgica Acta*.

[54] Kurtz JM, Spitalier JM, Amalric R (1988). Mammary recurrences in women younger than forty. *International Journal of Radiation Oncology Biology Physics*.

[55] Clark RM, Whelan T, Levine M (1996). Randomized clinical trial of breast irradiation following lumpectomy and axillary dissection for node-negative breast cancer: an update. *Journal of the National Cancer Institute*.

[56] Kim SH, Simkovich-Heerdt A, Tran KN, Maclean B, Borgen PI (1998). Women 35 years of age or younger have higher locoregional relapse rates after undergoing breast conservation therapy. *Journal of the American College of Surgeons*.

[57] Bantema-Joppe EJ, De Munck L, Visser O (2011). Early-stage young breast cancer patients: impact of local treatment on survival. *International Journal of Radiation Oncology Biology Physics*.

[58] Van Der Hage JA, Mieog JSD, Van De Velde CJH, Putter H, Bartelink H, Van De Vijver MJ (2011). Impact of established prognostic factors and molecular subtype in very young breast cancer patients: pooled analysis of four EORTC randomized controlled trials. *Breast Cancer Research*.

[59] Van’t Veer LJ, Dai H, Van de Vijver MJ (2002). Gene expression profiling predicts clinical outcome of breast cancer. *Nature*.

[60] Wang Y, Klijn JGM, Zhang Y (2005). Gene-expression profiles to predict distant metastasis of lymph-node-negative primary breast cancer. *The Lancet*.

[61] Van De Vijver MJ, He YD, Van ’T Veer LJ (2002). A gene-expression signature as a predictor of survival in breast cancer. *The New England Journal of Medicine*.

[62] El-Harith EHA, Abdel-Hadi MS, Steinmann D, Dork T (2002). *BRCA1* and *BRCA2* mutations in breast cancer patients from Saudi Arabia. *Saudi Medical Journal*.

[63] Robson ME, Gilewski T, Haas B (1998). BRCA-associated breast cancer in young women. *Journal of Clinical Oncology*.

[64] Levy-Lahad E, Friedman E (2007). Cancer risks among *BRCA1* and *BRCA2* mutation carriers. *British Journal of Cancer*.

[65] Peto J, Collins N, Barfoot R (1999). Prevalence of *BRCA1* and *BRCA2* gene mutations in patients with early- onset breast cancer. *Journal of the National Cancer Institute*.

[66] Sng JH, Chang J, Feroze F (2000). The prevalence of *BRCA1* mutations in Chinese patients with early onset breast cancer and affected relatives. *British Journal of Cancer*.

[67] Eerola H, Heikkilä P, Tamminen A, Aittomäki K, Blomqvist C, Nevanlinna H (2005). Relationship of patients’ age to histopathological features of breast tumours in BRCA1 and BRCA2 and mutation-negative breast cancer families. *Breast Cancer Research*.

[68] Althuis MD, Brogan DD, Coates RJ (2003). Breast cancers among very young premenopausal women (United States). *Cancer Causes and Control*.

[69] Navarro Silvera SA, Jain M, Howe GR, Miller AB, Rohan TE (2006). Energy balance and breast cancer risk: a prospective cohort study. *Breast Cancer Research and Treatment*.

[70] Lahmann PH, Hoffmann K, Allen N (2004). Body size and breast cancer risk: findings from the European Prospective Investigation into Cancer and Nutrition (EPIC). *International Journal of Cancer*.

[71] Coates RJ, Uhler RJ, Hall HI (1999). Risk of breast cancer in young women in relation to body size and weight gain in adolescence and early adulthood. *British Journal of Cancer*.

[72] Abrahamson PE, Gammon MD, Lund MJ (2006). General and abdominal obesity and survival among young women with breast cancer. *Cancer Epidemiology Biomarkers and Prevention*.

[73] Daling JR, Malone KE, Doody DR, Johnson LG, Gralow JR, Porter PL (2001). Relation of body mass index to tumor markers and survival among young women with invasive ductal breast carcinoma. *Cancer*.

[74] Berclaz G, Li S, Price KN (2004). Body mass index as a prognostic feature in operable breast cancer: the International Breast Cancer Study Group experience. *Annals of Oncology*.

[75] Petrelli JM, Calle EE, Rodriguez C, Thun MJ (2002). Body mass index, height, and postmenopausal breast cancer mortality in a prospective cohort of US women. *Cancer Causes and Control*.

[76] Protani M, Coory M, Martin JH (2010). Effect of obesity on survival of women with breast cancer: systematic review and meta-analysis. *Breast Cancer Research and Treatment*.

[77] Loi S, Milne RL, Friedlander ML (2005). Obesity and outcomes in premenopausal and postmenopausal breast cancer. *Cancer Epidemiology Biomarkers and Prevention*.

[78] Keegan THM, Milne RL, Andrulis IL (2010). Past recreational physical activity, body size, and all-cause mortality following breast cancer diagnosis: results from the breast cancer family registry. *Breast Cancer Research and Treatment*.

[79] Zhang S, Folsom AR, Sellers TA, Kushi LH, Potter JD (1995). Better breast cancer survival for postmenopausal women who are less overweight and eat less fat: the Iowa women’s health study. *Cancer*.

[80] Maru S, van der Schouw YT, Gimbrère CH, Grobbee DE, Peeters PH (2004). Body mass index and short-term weight change in relation to mortality in Dutch women after age 50 y. *The American Journal of Clinical Nutrition*.

[81] Morimoto LM, White E, Chen Z (2002). Obesity, body size, and risk of postmenopausal breast cancer: the women’s health initiative (United States). *Cancer Causes and Control*.

[82] Swanson CA, Coates RJ, Schoenberg JB (1996). Body size and breast cancer risk among women under age 45 years. *American Journal of Epidemiology*.

[83] Ursin G, Longnecker MP, Haile RW, Greenland S (1995). A meta-analysis of body mass index and risk of premenopausal breast cancer. *Epidemiology*.

[84] Van Den Brandt PA, Spiegelman D, Yaun SS (2000). Pooled analysis of prospective cohort studies on height, weight, and breast cancer risk. *American Journal of Epidemiology*.

[85] Peacock SL, White E, Daling JR, Voigt LF, Malone KE (1999). Relation between obesity and breast cancer in young women. *American Journal of Epidemiology*.

[86] Willett WC, Browne ML, Bain C (1985). Relative weight and risk of breast cancer among premenopausal women. *American Journal of Epidemiology*.

[87] Harvie M, Hooper L, Howell AH (2003). Central obesity and breast cancer risk: a systematic review. *Obesity Reviews*.

[88] Sonnenschein E, Toniolo P, Terry MB (1999). Body fat distribution and obesity in pre- and postmenopausal breast cancer. *International Journal of Epidemiology*.

